# Screening diagnosis of executive dysfunction after ischemic stroke and the effects of transcranial magnetic stimulation: A prospective functional near‐infrared spectroscopy study

**DOI:** 10.1111/cns.14118

**Published:** 2023-02-14

**Authors:** Yuanwen Liu, Jing Luo, Jie Fang, Mingyu Yin, Jie Cao, Shuxian Zhang, Li Huang, Qilin Cheng, Yinan Ai, Haiqing Zheng, Xiquan Hu

**Affiliations:** ^1^ Department of Rehabilitation Medicine The Third Affiliated Hospital of Sun Yat‐sen University Guangzhou China; ^2^ Department of Rehabilitation Medicine Xiamen Humanity Rehabilitation Hospital Xiamen China; ^3^ Department of Education Guangdong Provincial People's Hospital (Guangdong Academy of Medical Sciences), Southern Medical University Guangzhou China; ^4^ Department of Sport Rehabilitation Shanghai University of Sport Shanghai China

**Keywords:** executive dysfunction, fNIRS, functional connectivity, ischemic stroke, transcranial magnetic stimulation

## Abstract

**Background:**

Post‐ischemic stroke executive impairment (PISEI) is a serious obstacle for patients to returning to their society and is currently difficult to screen early and clinically ineffective.

**Aim:**

The aim of the study was to clarify whether functional near‐infrared spectroscopy (fNIRS) can be used as a rapid screening tool for PISEI and to explore the efficacy of transcranial magnetic stimulation (TMS) in PISEI patients and the changes in brain function.

**Methods:**

A single‐blind, randomized controlled study design was used to detect hemodynamic differences by fNIIRS in 16 PISEI patients and 16 healthy subjects during the resting state and Stroop task, respectively. After 3 days, all subjects received a single TMS intervention and underwent simultaneous fNIRS testing for the Stroop task before and 3 days after the TMS intervention.

**Results:**

PISEI patients had significantly higher HbO_2_ content in the left dorsolateral prefrontal cortex (DLPFC), the right pre‐motor cortex (PMC) and the right primary sensorimotor cortex (SM1) during the Stroop task compared to the resting state (*F* = 141.966, *p* < 0.001), but significantly lower than healthy subjects (*T* = −3.413, *p* = 0.002). After TMS intervention, PISEI patients' time and error number scores on the Stroop test were significantly enhanced, and the functional activity of the above‐mentioned brain regions was significantly more active than at baseline, while the strength of their functional connections with each other was markedly increased.

**Conclusions:**

fNIRS helped screen and diagnose PISEI. A single TMS session benefited PISEI patients with effects lasting 3 days, which may be attributed to activation of the left DLPFC, right PMC and right SM1 brain regions.

## Graphical abstract

fNIRS can be used as an auxiliary diagnostic tool for PISEI. TMS has a certain therapeutic effect on PISEI patients, and the effect can be maintained for 3 days, which may be attributed to the activation of left DLPFC, right PMC and SM1 brain regions and the enhancement of synergism of neural remodeling.

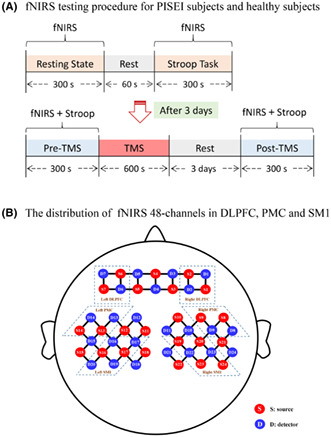

## 1 INTRODUCTION

Stroke is the leading cause of death and disability in China. Ischemic stroke is the most conspicuous type of stroke, and about 2/3 of patients with ischemic stroke have cognitive impairment.^1^ Executive dysfunction is among the primary manifestations of cognitive impairment after ischemic stroke. The main symptoms of post‐ischemic stroke executive impairment (PISEI) are diminished ability to initiate and plan, commonly characterized by decreased or loss of problem‐solving ability.^2^, ^3^ PISEI not only seriously affects patients' quality of life but also affects their work and study, social participation, and return to work.^4^, ^5^ Currently, early diagnosis of PISEI is difficult and effective interventions are lacking. Consequently, exploring technical methods to resolve the problems of difficult initial assessment and screening of PISEI and the poor efficacy of clinical treatment are the prevailing desperate problems in terms of PISEI rehabilitation.

The current clinical screening and diagnosis of PISEI relies predominantly on patients' clinical symptoms and neuropsychological scales, and lacks precise and quantitative evaluation indexes.^6^ Furthermore, the symptoms of PISEI patients are heterogeneous, and it is impossible to recognize PISEI prematurely and accurately only based on patients' symptoms and signs.^7^, ^8^ Although the neuropsychological scale can comprehensively evaluate multiple cognitive domains of subjects, it takes a long time and is easily affected by aphasia, the subjective will of the evaluator, the degree of cooperation of the patient and the level of education, which leads to great difficulties in completing the rapid screening and accurate diagnosis of PISEI.^9^ Therefore, it is particularly necessary to explore a fast, objective, and quantitative PISEI evaluation index. Functional near‐infrared spectroscopy (fNIRS) is a noninvasive brain functional neuroimaging technique that has been increasingly applied to the examination of brain diseases. A study has reported that fNIRS based on neurovascular coupling (NVC) effects can effectively distinguish patients with mild cognitive impairment from normal adults.^10^ Niu et al. detected functional deficits in the frontal and temporal cortex as well as working memory (WM) and cognitive impairment in MCI patients by fNIRS, suggesting that fNIRS may be a useful tool for assessing brain activation in patients with cognitive impairment.^11^ Jean et al. also found that fNIRS can characterize and monitor neurocognitive dysfunction in cancer patients through neurovascular coupling features.^12^ However, whether fNIRS can likewise reflects the cognitive function of PISEI patients and identifies the diagnosis of PISEI has not been reported in the literature and remains to be further explored and studied.

Currently, the clinical intervention of PISEI mainly focuses on cognitive function training. Although its effectiveness has been confirmed by some studies, it is time‐consuming and laborious, with slow onset effect, and its efficacy is easily affected by patients' compliance. However, PISEI patients often fail to cooperate in cognitive training due to their cognitive impairment, resulting in a significant reduction in the efficacy.^13^ Therefore, it is particularly important to explore an effective and feasible new PISEI rehabilitation technology. Transcranial magnetic stimulation (TMS) is an emerging neural regulation technology, and its application in PISEI rehabilitation has attracted more and more attention in recent years. Studies have shown that TMS has a promising therapeutic potential for post‐stroke cognitive impairment (PSCI)^14^ and our previous study also found that TMS is beneficial for improving PSCI,^15^, ^16^ which laid a solid foundation for the follow‐up of this study. However, overall there are relatively few studies on TMS for PISEI, resulting in its efficacy and exact recovery mechanisms and therapy ideas remaining undefined.

Here, we aimed to explore whether fNIRS can be used as a prompt diagnostic tool for PISEI and to clarify the efficacy of TMS interventions for PISEI and the changes in brain function. The results of this research will provide an empirical basis for the feasibility of fNIRS as a screening and diagnostic tool for PISEI, and also provide new therapeutic ideas for the possibility of TMS as a new technology to upgrade PISEI, which is of great significance to further optimize the intervention strategy of PISEI.

## 2 METHODS

### 2.1 Study design and participants

This study involved a single‐blind, randomized controlled trial based on the guidelines of the Declaration of Helsinki. The study was registered at Chictr.org (Chinese Clinical Trial Registry: ChiCTR1900025467) on August 27, 2019, and approved by the Clinical Medical Research Ethics Committee of the Third Affiliated Hospital of Sun Yat‐sen University (ID: [2016]2‐6). This research was conducted at the Department of Rehabilitation Medicine, the Third Affiliated Hospital of Sun Yat‐sen University, Guangzhou, China from December 2019 to December 2021. Informed consent was obtained from subjects to experiment with our study, and a total of 32 subjects were eventually enrolled in this subject, including 16 PISEI subjects and 16 healthy subjects.

The inclusion criteria for PISEI subjects were as follows: (1) diagnosed with ischemic stroke; (2) scored 10–27 on Mini‐mental State Examination (MMSE) and scored below 26 on Montreal Cognitive Assessment (MoCA); (3) scored <4 out of five on MoCA's visuospatial and executive function subtests; (4) in stable condition and can complete paper and pencil tests; (5) first onset and course of disease (6–18 months); (6) aged 45–75 years; and (7) capable of providing informed consent for the study. Healthy subjects were included if they (1) aged 45–75 years; (2) no abnormalities on cranial fMRI examination; (3) MMSE score ≥ 27 and MoCA score ≥ 26; and (4) not taking medications. PISEI and healthy subjects were excluded if they had metal implants, a history of other neurological disorders, acute cardiopulmonary dysfunction, multi‐organ failure, brain tumors, psychiatric disorders, history or family history of seizures, complete aphasia, color blindness, or deafness, and had previously done cognitive tests.

### 2.2 Randomization and blinding

All eligible PISEI and healthy subjects were randomly assigned to the resting state, Stroop task of fNIRS simultaneous testing and TMS intervention. A computer‐generated randomization table with a 1:1 ratio, created by a statistician not involved in the research, was used to randomly allocate patients to the above program. The distribution method was assigned confidentiality through sealed opaque envelopes, and the envelopes were kept in a central, closed closet by independent researchers who were not involved in the study. After the initial evaluation, the allocation scheme was disclosed to the therapist operating the TMS. In order to make the gender balance between PISEI patients and healthy subjects more balanced, we stratified and randomized the above subjects according to gender. A blinded practitioner assessed the participants at baseline and 3 days after the end of the TMS intervention, and the data were statistically analyzed by a physician who did not know the grouping status.

### 2.3 TMS program

All subjects received a single high‐frequency TMS intervention. TMS was administered using a magnetic stimulation device (CCY‐IA Wuhan Yiruide Co., Ltd.), attached to a focal figure‐of‐eight shape coil (each loop had a diameter of 3.5 cm). The coil was systematically displaced (mapping) over the subject's primary motor cortex until the largest consistent motor evoked potential response from the contralateral first dorsal interosseous was recorded. The resting motor threshold (RMT) of the first interosseous was defined as the minimum intensity, which elicited a motor evoked potential of 50 μV in at least five out of 10 consecutive treatments. In our study, the stimulation target was the left DLPFC, and a navigation (Visor 2™ LT, Solutions GmbH Manufacturer, USA) was used for cranial localization. The specific localization process was as follows: the subject was seated in a reclining chair with armrests of moderate height, facing the navigation infrared spectral motion tracking system, followed by coil correction and creation of an individualized head model, which was matched to the subject's skull, and then the stimulation target (left DLPFC) was located and marked based on the subject's MRI image, completing the localization operation. Each subject received only one TMS intervention at an intensity of 90% of the resting motor threshold (RMT) at a frequency of 10 Hz, with 700 pulses and a stimulation duration of 10 min. To assess the safety of the TMS intervention in this study, we used the dizziness handicap inventory (DHI) to evaluate subjects for adverse effects, including dizziness, headache, and balance disorders during standing and walking.

### 2.4 Resting state and Stroop task detection for fNIRS

**FIGURE 1:**
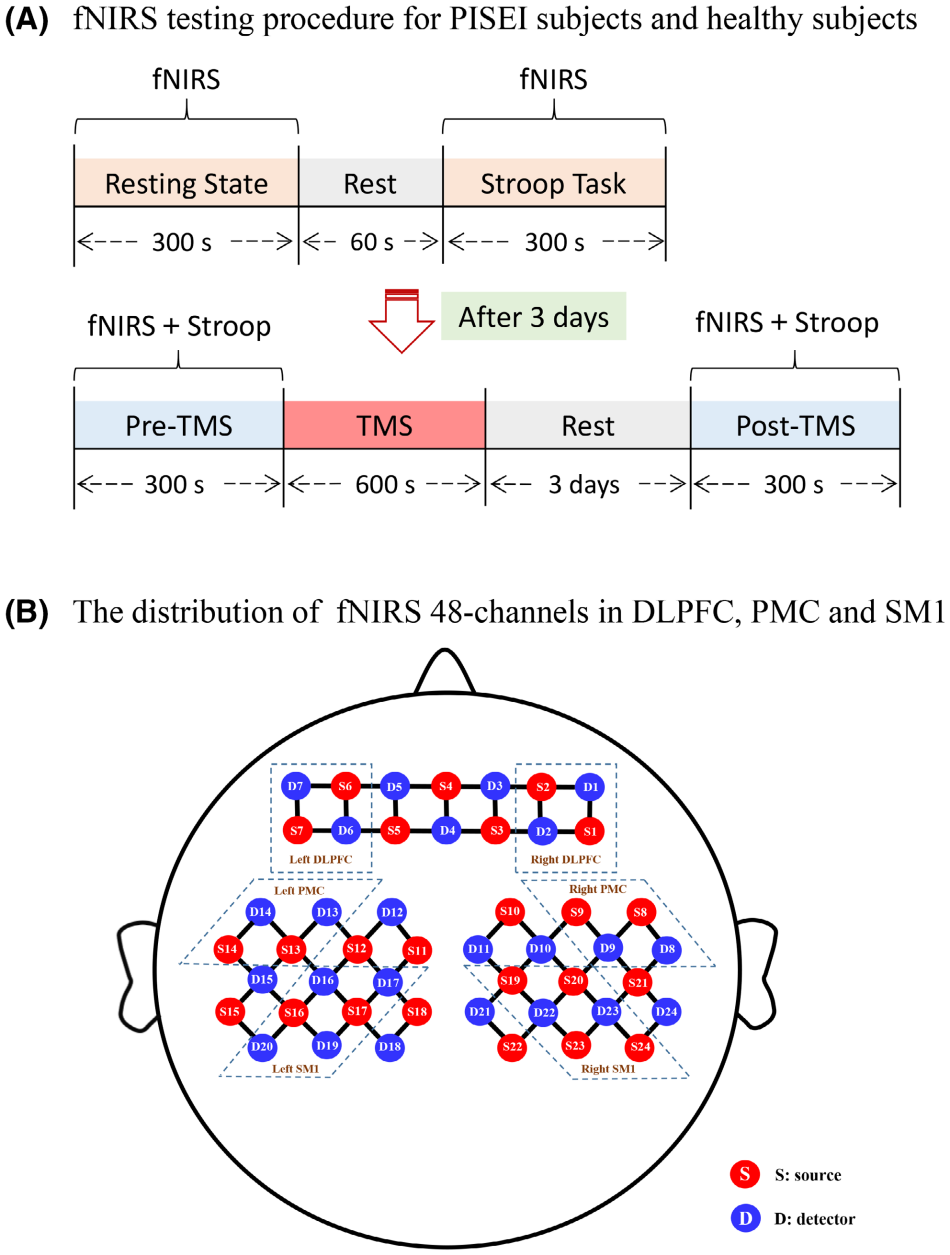
fNIRS testing procedure and fNIRS 48 channels distribution map. (A) fNIRS testing procedure for PISEI subjects and healthy subjects. (B) The distribution of fNIRS 48‐channels in DLPFC, PMC, and SM1. DLPFC, dorsolateral prefrontal cortex; fNIRS, functional near‐infrared spectroscopy; PMC, pre‐motor cortex; SM1, primary sensorimotor cortex. TMS, transcranial magnetic stimulation.

All PISEI patients and healthy subjects underwent synchronous fNIRS testing in the resting and Stroop task state, respectively. The specific testing procedure was as follows: (1) first 300 s of resting‐state detection of fNIRS, followed by 60 s of rest, and then 300 s of Stroop task state detection; (2) 3 days later, 300 s of Stroop task state detection before TMS intervention, followed by 600 s of TMS intervention, and then 300 s of Stroop post‐TMS task state detection (details in Figure 1A).

The Stroop color‐word task test consisted of cards A, B, and C. Card A had the words “red, yellow, blue, and green” written in black letters and required the participant to read the words as rapidly and precisely as possible. Card B had the circles of yellow, blue, red, and green printed on it and required the participant to read the names of the colors as quickly and correctly as possible. Card C was written in yellow, blue, green, and red with four words “green, red, blue, and yellow,” the colors and words did not match, and the subjects were asked to read the names of colors as swiftly and accurately as possible. The shorter the time and the lower the number of errors, the better the executive function.^17^

### 2.5 fNIRS detection procedure

A multi‐channel desktop NirScan fNIRS device (Huichuang. China) was used to collect the subjects' resting and Stroop task state data in a quiet environment, and the changes in oxygenated hemoglobin (HbO_2_), deoxygenated hemoglobin (HbR), and total hemoglobin (HbT) signals were recorded. NirScan encompasses 24 pairs of transmitting and receiving poles, which can recognize 48 channels of signals in the whole brain. The sampling frequency was 11 Hz and the wavelength was 730 nm and 850 nm. The coordinates of each optical pole on the Montreal Neurological Institute (MNI) brain template were determined using the 10/20 international standard lead system, combined with the coordinate positioning function of the SPM software, with reference to the coordinates and optical pole positions. The regions of interest (ROI) in this study were the left/right dorsolateral prefrontal cortex (DLPFC), left/right pre‐motor cortex (PMC), and left/right primary sensorimotor cortex (SM1), as demonstrated in Figure 1B.

### 2.6 Data processing

Data processing was executed in MATLAB (R2017a, Mathworks Inc.). The motion artifacts were removed by spline interpolation, and the raw light intensity was converted to changes in the concentration of HbO_2_ and HbR using the modified Beer–Lambert law. To extract the drift and heartbeat interference of HbO_2_ and HbR, we utilized an optimal filter with a finite impulse response of order 1000. We applied a multichannel short‐channel regression (SCR) based on non‐negative least squares (GLM^multiSS^) to minimize the impact of physiological variations to obtain better anti‐interference effects. The validation of signal quality of short‐range channels followed the approach applied by Perdue et al.^18^ The band power of HbO_2_ (0.07–0.14 Hz) and its pulse band power (0.6–2 Hz) were normalized to retrieve its Mayer wave (MW) amplitude and the median value of all long‐range channels was extracted. *T*‐values were stored in a vector, and the average *t*‐values for the corresponding channel were used for ROI analysis.

### 2.7 Statistical analysis

The software SPSS version 26.0 (IBM Corp) was used for statistical analysis. Baseline data such as demographics and clinical characteristics were balanced and comparable. All the subjects who underwent randomization were included in the primary and exploratory analyses, and data for subjects who completed or discontinued the trial without an outcome were excluded from the day of their last visit. Events that occurred after that visit were not included. We used a mixed model for repeated measurements to estimate mean differences in HbO_2_ values and Stroop test assessment outcomes between groups, with adjustment for baseline covariates.

Shapiro–Wilk normality test was used to evaluate data distribution for all continuous variables. If the continuous variables obeyed normal distribution with equal variance were expressed as mean and standard deviation (SD), paired‐sample *t*‐test was used for intra‐group comparison and independent samples *t*‐test was used for inter‐group comparison. Repeated measures analysis of variance (ANOVA) was used to compare the HbO_2_ values and clinical scale assessments of the two groups of subjects at different time points, and further post hoc multiple comparisons were performed using the Bonferroni correction if the differences were statistically significant. The continuous variables that did not comply with the normal distribution were expressed in median and interquartile ranges, and a nonparametric rank‐sum test was used for intra‐ and inter‐group comparisons. Categorical variables were summarized with frequencies and percentages and were analyzed with the Chi‐square test. Estimated mean group differences was reported with 95% CIs and 2‐sided *p* values, with values <0.05 considered significant, and false discovery rate (FDR) was corrected.

## 3 RESULTS

### 3.1 Demographic and clinical characteristics

**FIGURE 2:**
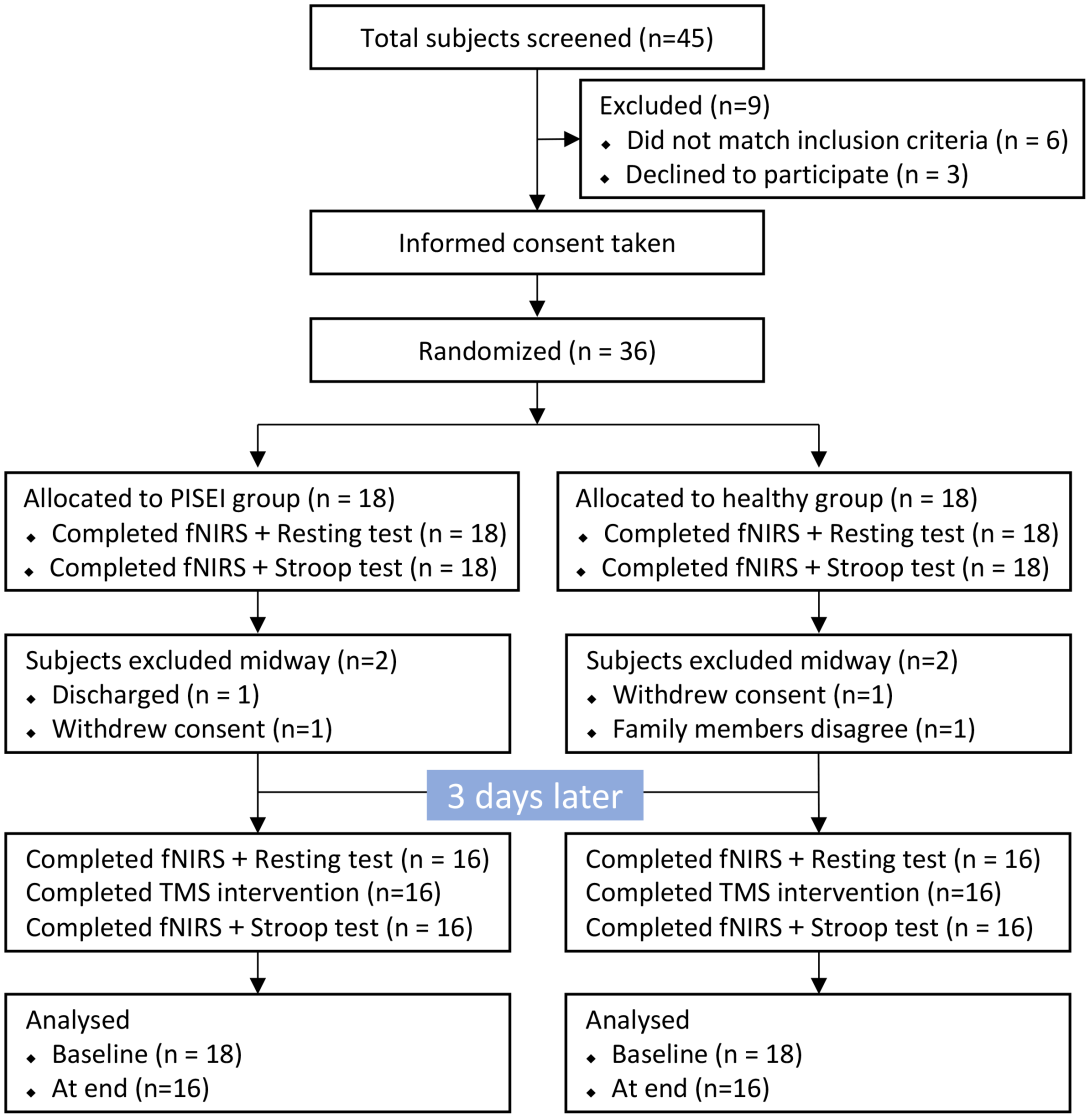
CONSORT flowchart

The study flow diagram is demonstrated in Figure 2 and Table 1 summarizes the demographic and baseline clinical features of all study participants. A total of 16 participants, with a mean age of 54.38 years, ten males and six females, were enrolled in the PISEI group. A total of 16 participants, with a mean age of 55.69 years, eight males and eight females, were enrolled in the Healthy group. No significant differences in baseline characteristics such as age, sex, and education degree were noted between the two groups. Clinical information on stroke in healthy subjects matched to subjects in the PISEI group was not gathered because they were in excellent physical condition. No adverse reactions were reported throughout the fNIRS and TMS studies.

**TABLE 1:** Demographics and baseline clinical characteristics of all subjects

Characteristics	PISEI subjects (*n* = 16)	Healthy subjects (*n* = 16)	*p* value
Age (years, Mean ± SD)	54.38 ± 8.48	55.69 ± 6.95	0.636^a^
Sex, *n* (%)			0.476^b^
Male	10 (62.5%)	8 (50%)	
Female	6 (37.5%)	8 (50%)	
Education degree (years, Mean ± SD)	9.44 ± 3.79	10.12 ± 4.38	0.639^a^
Ischemic stroke onset (months, Mean ± SD)	3.94 ± 1.81	—	—
Affected hemisphere, *n* (%)			
Right	9 (56.25%)	—	—
Left	7 (43.75%)	—	—
Stroop test			
Times (seconds, Mean ± SD)	161.54 ± 25.08	72.02 ± 22.63	<0.001^a^
Errors (numbers, Mean ± SD)	10.88 ± 2.55	3.56 ± 1.46	<0.001^a^
HbO_2_ value (kg/L, Mean ± SD)	0.17 ± 0.03	0.24 ± 0.03	<0.001^a^
FC coefficient (Mean ± SD)	0.18 ± 0.03	0.25 ± 0.03	<0.001^a^
Coefficient threshold (Mean ± SD)	14.71 ± 3.00	23.44 ± 3.68	<0.001^a^

Abbreviations: %, percentages; FC, functional connection; *n*, frequency; SD, standard deviation.

^a^ Analyzed by independent sample *t*‐test.

^b^ Analyzed by *χ* ^2^ test.

### 3.2 Changes in fNIRS detection results of resting‐state and Stroop task state before TMS intervention

**FIGURE 3:**
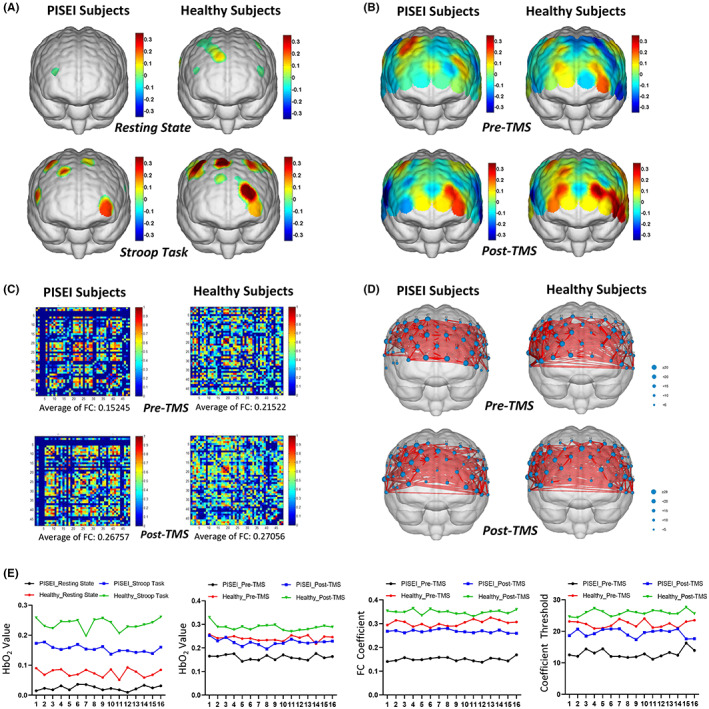
fNIRS activation topographic maps for all subjects. (A) Brain averaged activation maps from changes in HbO_2_ for all subjects during the resting state and the Stroop task. (B) Brain averaged activation maps from changes in HbO_2_ for all subjects during the Stroop task before and after TMS. (C) Mean functional connection strength matrix for all subjects during the Stroop task before and after TMS. (D) Brain network connection coefficient thresholds for all subjects during the Stroop task before and after TMS. (E) Comparisons of average HbO_2_ values, functional connection coefficients and brain network connection coefficient thresholds for all subjects. FC, functional connection; HbO_2_, oxygenated hemoglobin; TMS, transcranial magnetic stimulation.

Compared to the resting state, the main effect of the amount of change in HbO_2_ values was markedly higher in the left DLPFC, the right PMC and the right SM1 during the Stroop task in PISEI patients (*F* = 141.966, *p* < 0.001). Besides, PISEI patients had significantly lower main effects for the amount of change in HbO_2_ values in the bilateral DLPFC, right PMC and right SM1 during the Stroop task than healthy subjects (*T* = −3.413, *p* = 0.002). The above results are displayed in Figure 3A.

### 3.3 Changes in fNIRS and scale assessment results for Stroop task states before and after TMS intervention

After TMS intervention, the main effect of HbO_2_ value of left DLPFC, right PMC, and right SM1 in PISEI patients (*T* = −8.204, *p* < 0.001; Figure 3B), the main effect of functional connection coefficient (*T* = −10.493, *p* < 0.001; Figure 3C), the main effect of the variation of brain network connection coefficient threshold (*T* = −9.088, *p* < 0.001; Figure 3D) were significantly higher than that before TMS intervention. The specific data results of fNIRS for the above two types of subjects are disclosed in Figure 3E.

In terms of the Stroop color‐word test performance, the time and number of errors scores of Stroop test in PISEI patients and healthy subjects improved significantly after the TMS intervention compared with the pre‐TMS intervention (*p* < 0.001), with PISEI patients showing better improvement in the number of errors scores of Stroop test than healthy subjects (*p* < 0.001). However, there was no significant difference in the improvement of time score of Stroop test between the two categories of subjects. The specific statistical values of the above Stroop color‐word test can be seen in Table 2.

**TABLE 2:** Changes in Stroop performance of all subjects before and after TMS intervention

Features	PISEI subjects (*n* = 16)			Healthy subjects (*n* = 16)			Between‐group difference in change, Mean (95% CI)	*p* Value**
	Pre‐TMS, Mean ± SD	Post‐TMS, Mean ± SD	Change, Mean (95% CI)	Pre‐TMS, Mean ± SD	Post‐TMS, Mean ± SD	Change, Mean (95% CI)		
Stroop‐Times, Seconds	161.54 ± 25.08	134.26 ± 18.74	27.28 (17.59 to 36.97)	72.02 ± 22.63	42.85 ± 17.52	29.16 (25.29 to 33.04)	1.88 (−8.17 to 11.93)	0.704
*p* Value*		<0.001			<0.001			
Stroop‐Errors, Numbers	10.88 ± 2.55	6.31 ± 1.89	4.56 (3.98 to 5.15)	3.56 ± 1.46	0.94 ± 1.06	2.63 (2.01 to 3.24)	1.94 (1.19 to 2.68)	<0.001
*p* Value*		<0.001			<0.001			

Abbreviations: CI, confidence intervals; SD, standard deviation; TMS, transcranial magnetic stimulation.

* *p* value within groups.

** *p* value between groups.

## 4 DISCUSSION

This single‐blind, randomized controlled design trial aimed to explore the differences in brain activity between patients with PISEI and healthy subjects during the execution of intricate cognitive activities by fNIRS, to provide an empirical basis for the feasibility of fNIRS as a rapid identification, diagnostic tool for PISEI, and in addition to exploring the efficacy of TMS on executive function in patients with PISEI and the changes in brain function. Specifically, we used fNIRS, a non‐invasive brain imaging technology, to monitor the hemoglobin content and hemodynamic differences between PISEI patients and healthy adults during the Stroop task in real‐time and dynamically, respectively, to summarize and analyze the altering patterns of neurovascular coupling effects in the brain regions of the two types of subjects. On the other hand, TMS with a frequency of 10 Hz was used to stimulate the left DLPFC of all subjects in a single session. The hemodynamic differences in the above subjects during the execution of Stroop task before and after TMS intervention were detected in real‐time by fNIRS to uncover the efficacy and the changes in brain function of TMS therapy of executive function in patients with ischemic stroke. The results of 32 eligible subjects who completed this protocol illustrated that PISEI patients had significantly higher activation of brain areas during the execution of Stroop task than in the resting state, but significantly lower than in healthy subjects. Moreover, PISEI patients with the high‐frequency TMS intervention indicated significant improvements in executive function, significantly higher activation of brain areas than before the intervention, and significantly greater functional connectivity of brain areas with each other. The implications of these results will be discussed below.

### 4.1 Differences between PISEI subjects and healthy subjects in resting‐state and Stroop task state respectively

In this study, the fNIRS‐based analysis disclosed that the oxyhemoglobin content in the left DLPFC, the right PMC and the right SM1 was markedly heightened in PISEI patients during the Stroop task compared with the resting state. Nevertheless, the oxyhemoglobin concentrations in these brain regions were considerably lower than those in healthy subjects, implying that the occurrence of PISEI may be related to the suppression of blood oxygen metabolism and functional activity in the DLPFC, PMC and SM1 brain regions. These results may be because DLPFC is a susceptible area for memory, attention, and control, which are closely related to cognitive functions,^19^ and PMC and SM1 brain regions are related to executive functions such as execution, inhibition, and fixation switching,^20^ so the above brain regions will show a certain activation state when PISEI patients perform complex cognitive tasks. It is worth noting that the results of this study also indicate that fNIRS has the potential to detect differences in brain function between patients with PISEI and healthy subjects, thus contributing to timely screening and diagnosis of PISEI. Other studies have reported differences in cerebral hemodynamics between PSCI patients and healthy subjects.^21^ Enhanced cognitive task difficulty elicits stronger neurovascular coupling responses in healthy young adults.^22^ This proposes that neurovascular coupling damage may play an important role in the pathogenesis of PSCI and that performing complex cognitive tasks may considerably promote neurovascular coupling effects in brain regions of healthy young adults. The above findings are comparable to the results of the present study, but the subjects of the above study were healthy young adults, and it is still unknown whether similar results occur for PISEI. The present study complements the above studies to some extent and provides an experimental basis for understanding the mechanism of PISEI occurrence and fNIRS as an auxiliary diagnostic tool for PISEI.

It is still difficult to screen and diagnose PISEI in clinical practice, and its diagnosis primarily relies on patients' clinical symptoms and neuropsychological scales, which lack precise and quantitative evaluation indexes. Additionally, the scales used to assess PISEI patients are readily affected by factors such as aphasia, patients' literacy and collaboration in the assessment process, making it extremely difficult to complete prompt screening and accurate diagnosis of PISEI.^13^ Our study observed the differences in brain activation between PISEI patients and healthy subjects during the execution of Stroop task and the respective regular characteristics of fNIRS, which provides some experimental basis for the feasibility of fNIRS as a rapid diagnostic tool for PISEI and has some implications for solving the current problem of difficult early screening of PISEI.

Compared with traditional clinical scales, fNIRS has the following advantages as a screening and diagnostic tool for PISEI: (1) swift and handy: fNIRS can construct a predictive model for PISEI by obtaining cerebral hemodynamic data from PISEI patients and healthy adults, and incorporating intelligent algorithms such as big data and machine learning to recognize PISEI swiftly and conveniently at an early stage.^23^ (2) precise quantification: fNIRS can rapidly target the brain regions of PISEI patients by infrared light and characterize their blood oxygen metabolism, reflecting the cognitive function of patients by precisely quantifying the concentration of oxyhemoglobin.^24^ (3) real‐time visualization: fNIRS can observe the changes in hemoglobin content and hemodynamics of PISEI patients in real time and dynamically. The detection process is clearly visible, and intelligent presentation and adaptive adjustment of evaluation parameters can be achieved according to the feedback data of dynamic monitoring of brain function.^25^ And (4) guidance for follow‐up therapy: fNIRS can observe the hemodynamic changes in brain target areas in real time, dynamically and visually to infer the neural activity, and provide real‐time efficacy feedback and exact intervention guidance for the rehabilitation treatment of PISEI patients with the help of neurovascular coupling mechanism and blood oxygen metabolism in local brain areas.^26^

### 4.2 Therapeutic effects of TMS on PISEI patients and the changes in brain function

In this study, PISEI patients were stimulated once by TMS with a frequency of 10 Hz. Interestingly, we discovered that after TMS intervention, the Stroop test performance of PISEI patients was markedly better, executive function was significantly enhanced and the activities of left DLPFC, right PMC, and right SM1 brain regions were significantly active compared with those before TMS intervention. At the same time, the strength of the functional connection between them was also substantially improved, demonstrating that high‐frequency TMS may have a favorable effect on PISEI patients, which may be related to the activation of left DLPFC, right PMC, and right SM1 brain regions and the enhancement of synergistic neural remodeling. Our previous study showed that DLPFC stimulation with high‐frequency TMS can enhance the overall cognitive function, attention, memory, and executive function of patients with post‐stroke cognitive impairment, which has a certain synergistic effect on cognitive training therapy, and hints that this may be related to changes in the activity of frontal cortex.^15^, ^16^ However, the specific brain neural circuits and the characterization of neurophysiological activities remain to be further studied. The present study is a continuation and in‐depth of preceding studies and accumulates evidence‐based medical evidence to confirm that TMS can effectively intervene in PISEI and explore its neurological recovery mechanisms.

Others reported that stimulation of the DLPFC by high‐frequency TMS can substantially enhance the cognition, memory function, working memory, attention function, and emotional state of PSCI patients.^27^, ^28^, ^29^ Low‐frequency TMS stimulation of contralateral DLPFC can effectively promote the recovery of attention function in PSCI patients,^30^ and intermittent theta‐burst stimulation (iTBS) can improve the symptoms and cognitive function of Alzheimer's disease,^31^ which is consistent with our findings. Unlike the studies mentioned above, the present study was limited to the specific disease of PISEI, which is more targeted, and not all of the studies mentioned above conducted TMS targeting by navigation systems, and no relevant brain function changes have been explored. Based on this, we used high‐frequency TMS based on a navigation system to intervene in PISEI patients and detected the changes of neurovascular coupling effects in relevant brain regions after TMS intervention in PISEI patients by fNIRS in real time, objectively and visually, which laid a solid foundation for revealing the changes in brain function of TMS improvement in PISEI.

As we all know, the accuracy and targeting of TMS localization is an important guarantee for the effectiveness of PISEI intervention. Otherwise, it is easy to “miss” the target. In this work, we utilized an infrared optics‐based navigation system for TMS localization, which can promptly and precisely locate the brain target area and overcome the shortcomings of conventional over‐reliance on operator experience. In addition, we used TMS‐compatible fNIRS to monitor the hemodynamic changes of local brain regions in real time and dynamically to infer the neural activity and provide real‐time feedback and intervention guidance for rehabilitation treatment. Current studies have also guided TMS interventions in cognitive function through fNIRS. For example, Curtin et al.^32^ used fNIRS to detect changes in activity in the corresponding brain regions of subjects after TMS intervention and found a significant increase in the neurological efficiency of cognitive processing speed. Still, the stimulation targets of TMS in these studies were widespread and not navigated, and the subjects were healthy participants, so its effectiveness in PISEI patients is still unknown. This research, based on fNIRS‐guided TMS intervention for PISEI, certified the therapeutic effect of TMS on PISEI, and recognized the potential neural mechanism that this is related to the activation of the left DLPFC, right PMC and right SM1 brain regions and their synergistic enhancement of neural remodeling, providing a new therapeutic idea and theoretical basis for TMS clinical intervention for PISEI.

### 4.3 Study significance and limitations

To our knowledge, this is the first single‐blind randomized controlled trial that investigated real‐time, dynamic monitoring of hemodynamic differences between PISEI subjects and healthy subjects employing fNIRS during the execution of Stroop test in both types of subjects, followed by a selection of stimulation targets for TMS intervention in PISEI based on fNIRS data and precise treatment with TMS guided by a navigation system. Although our work is a great contribution to exploring the feasibility of fNIRS as a prompt screening diagnostic tool for PISEI and revealing TMS to improve the brain function changes in PISEI, there are some limitations. For example, this was only a single‐center preliminary study with a small sample size and lack of follow‐up, which failed to track the long‐term effectiveness of TMS in PISEI patients. In addition, although we applied fNIRS as a neuroimaging technique for efficacy evaluation, we only gathered behavioral data on executive function through the Stroop test, which failed to systematically assess executive function comprehensively in terms of behavior.

## 5 CONCLUSION

PISEI may be related to suppressing of blood oxygen metabolism and functional activities in DLPFC, PMC, and SM1 brain regions. fNIRS, based on dynamic monitoring of hemodynamics, can be used as an auxiliary tool for prompt screening and diagnosis of PISEI. A single TMS session was favorable for PISEI patients, and the efficacy lasted up to 3 days, which may be associated with the activation of left DLPFC, right PMC, and right SM1 brain regions and the enhanced synergism of neural remodeling. The TMS stimulation parameters and treatment environment in our study did not provoke adverse reactions in the subjects, and the safety of the above treatment conditions was controllable under the specific conditions in this research. In future work, randomized controlled trials with large samples, multi‐centers, and follow‐up sessions should be carried out, and systematic and varied behavioral evaluation indicators should be adopted to comprehensively validate the exact efficacy of TMS on PISEI.

## FUNDING INFORMATION

National Natural Science Foundation of China, Grant/Award Number: 81972151, 82172546, and 81871847; Guangdong Basic and Applied Basic Research Foundation, Grant/Award Number: 2019A1515011106 and 2017A030313586; National Natural Science Foundation of China Incubation Special Youth Program of the Third Affiliated Hospital of Sun Yat‐sen University, Grant/Award Number: 2021GZRPYQN10; National Key R&D Program of China, Grant/Award Number: 2018YFC2001603.

## CONFLICT OF INTEREST STATEMENT

All authors reported no conflicts of interest.

## Data Availability

The datasets generated during this study are available from the corresponding author upon reasonable request.
